# Differential expressions of miR-223, miR-424, miR-145, miR-200c, miR-139 in experimental rat chronic pancreatitis model and their relationship between oxidative stress, endoplasmic reticulum stress, and apoptosis

**DOI:** 10.22038/ijbms.2021.57664.12823

**Published:** 2021-09

**Authors:** Esra Guzel Tanoglu

**Affiliations:** 1 University of Health Sciences Turkey, Institution of Medical Sciences, Department of Molecular Biology and Genetics, Istanbul, Turkey; 2 University of Health Sciences Turkey, Experimental Medicine Research and Application Center, Uskudar, 34662, Istanbul, Turkey

**Keywords:** Apoptosis, Chronic pancreatitis, MicroRNAs, Oxidative stress, Quantitative real-time - polymerase chain reaction, Rat

## Abstract

**Objective(s):**

This study aimed to research the roles of miR-139, miR-221, miR-200c, miR-145, miR-223, miR-424, and miR-377 in endoplasmic reticulum stress (ERS), oxidative stress (OS), fibrosis, and apoptosis processes in chronic pancreatitis (CP) rat model.

**Materials and Methods:**

Fourteen rats were randomized into 2 groups (Group 1, sham group (n=7) and Group 2, CP group (n=7)). TGF-beta and malondialdehyde concentrations were measured in rat blood samples. qRT-PCR was used to investigate the expression levels of 7 miRNAs in the pancreas tissues. The correlations of mRNA undergoing significant changes with inflammation (TNF-α, IL-6), ERS (*Ire1-α, Perk*), apoptosis (*Caspase 3, Bcl-2*), OS (*Cat, Gpx1*), and fibrosis (*α-Sma*) were investigated***.***

**Results:**

The biochemical results and histopathological scores in Group 1 were statistically significantly high compared with Group 2 (*P*<0.5). Expression levels of seven miRNAs (miR-200c, miR-145, miR-223, miR-424) were significantly higher, while miR-139 was significantly lower in CP. In our study, we found that miR-200c, miR-145, and miR-139 may contribute to CP progression and cellular processes based on the correlation between ERS, OS, apoptosis, and inflammation with miRNA expression levels.

**Conclusion:**

miR-200c, miR-145, miR-139, miR-223, and miR-424 play roles in the CP model. They may be used as candidate biomarkers for the CP process.

## Introduction

Chronic pancreatitis (CP) is a fibroinflammatory disease-inducing parenchymal injury through intensification of recurrent pancreatitis attacks with observed exocrine and endocrine failure. Patients with CP are faced with short life expectancy due to frequent occurrence of other systemic diseases and a high chance of developing pancreas cancer (1). Genetic and environmental factors are included in the pathogenesis of CP. Currently, though there are studies illuminating the physiopathology of CP, the molecular mechanisms are not sufficiently understood (2, 3).

MicroRNAs (miRNA) are single change, encoding, endogenously-synthesized short RNA with nearly 18-24 nucleotide length. MiRNAs are deregulated in many diseases and cancer types and affect targeted genes (4, 5).

MiRNAs are very effective biomarkers in a variety of diseases, cancer, and for various cellular functions (6). Mature miRNAs are not complex, do not undergo known modification, have easily detectable expression levels, have tissue-specific expression profiles and arrays have high preservation levels between human and model organisms, in addition to very high stability, which makes miRNA ideal biomarkers (7, 8).

Considering miRNA research, certain miRNAs are proposed to be associated with inflammation, oxidative stress, and endoplasmic reticulum stress. These stages are present in the CP development process, though there are insufficient numbers of miRNA studies about CP (9). 

Oxidative stress increases oxidative injury by reducing anti-oxidant capacity in CP patients and contributes to development of CP (10). Similarly, endoplasmic reticulum stress increases in CP situations and induces pancreas injury via the pathologic calcium signal pathway (11). Oxidative stress and endoplasmic reticulum stress are important pathological mechanisms that require research in CP. 

In this study, we aimed to research the correlation between the variations in expression levels of miRNAs, identified with the *in silico* method, miR-139, miR-221, miR-200c, miR-145, miR-223, miR-424, and miR-377 with inflammation, endoplasmic reticulum, oxidative stress, and apoptosis progression in the CP process. Additionally, we aimed to investigate whether the miRNAs studied in an experimental CP rat model were new candidate biomarkers for disease diagnosis and treatment or not. 

## Materials and Methods

***Rat groups***

This study was conducted with the approval of the Hamidiye Local Ethics Committee for Animal Experiments of the University of Health Sciences, Turkey. Fourteen Sprague Dawley male rats weighing 250–300 g were used. The rats were housed in a 12-hour day-night cycle in cages kept at nearly 24 °C. The rats had free access to water and food, with food intake stopped 12 hr before the sacrifice of animals. The animals were randomized into two groups. 

Group 1: The sham group (n=7). One cubic centimeter of physiological serum was injected IP twice per day, 3 days per week for 6 weeks. 

Group 2: The CP group (n=7). To induce CP, 50 mcg/kg caerulein was injected IP, twice per day at one-hour intervals, 3 days per week (Monday, Wednesday, Friday) for 6 weeks. 

***Biochemical investigation***

Twelve hours after the final injections, rats were sacrificed under anesthesia and intracardiac blood samples were taken. The malondialdehyde (MDA) and transforming growth factor-beta (TGF-β) levels in blood samples were measured colorimetrically/fluorometrically with solid-phase sandwich enzyme-linked immunosorbent assay (ELISA) kits (Bioassay Technology Laboratory, USA) to determine oxidative stress. Measurements were completed according to the manufacturer’s protocols. 

***Histopathological investigation***

After sacrifice, the pancreas tissue was removed from rats, fixed in 10% formaldehyde, and then submerged in paraffin. Tissues were analyzed with a light microscope after hematoxylin-eosin staining to assess pancreas injury. Pathologic samples were assessed with a scoring system using inflammation and atrophy, and fibrosis scores for pancreas tissue were scored from 0 to 3 after making a modification (12).

***RNA isolation***

RNA isolation was performed from the pancreas tissue samples using MasterPure™ Complete DNA and RNA Purification Kit (Lucigen, USA) according to the manufacturer’s protocol. The purity and concentration values for RNA samples were measured with a Denovix DS-11 (DeNovix Inc., Wilmington, DE) spectrophotometer. 

***miRNA selection and qRT-PCR analysis***

After *in silico* analysis, miRNAs were selected using software programs such as DIANA TOOLS - mirPath v.3, miRWalk, miRPathDB, HMDD v3.0, followed by literature review. During these selections, it was preferred that the expression levels of miRNAs were changed in inflammation, pancreatic cancer, and/or pancreatitis.

The selected miRNAs’ (miR-139, miR-221, miR-200c, miR-145, miR-223, miR-424, and miR-377) in pancreatic tissue expression levels were analyzed with a Light Cycler Roche 480 II device using Taqman primers and TaqMan Universal PCR Master Mix (Thermo Fisher Scientific, USA). RNU43 was used as an internal control. Samples were studied with three repeats. 

***cDNA and qRT-PCR***

RNA samples obtained from tissues in CP and sham groups had cDNA synthesis completed to determine inflammation levels (TNF-α and IL-6), endoplasmic reticulum stress (*Ire1-α* and *Perk*), apoptosis levels (*Casp3, Bcl-2*), oxidative stress levels (*Cat* and *Gpx1*), and fibrosis (*α-Sma*). cDNA analysis was performed in line with the manufacturer’s instructions with a Transcriptor High Fidelity cDNA (Roche, Switzerland). Quantitative analysis of the expression levels of the stated genes was completed with real-time polymerase chain reaction using Roche’s SYBR Green Master Mix and a LightCycler480-II PCR (Roche, Switzerland). SYBR Green qRT-PCR was performed at 1 cycle of 95 °C for 5 min followed by 40 cycles of 95 °C for 10 sec, and 60 °C for 1 min. *Gapdh* was used as internal control and samples were studied with three repeats. The list of primers used in the study is given in Table 1.

***Statistical analysis***

Relative quantitation analysis for the results of real-time PCR experiments was performed by using the delta-delta-CT method. The data analysis was done by SPSS 20. *P*-values less than 0.05 were considered statistically significant. For the expression of the variations in the dataset, mean and standard deviation were calculated. The observations were normally distributed among all gene groups. For this reason, the means of two independent groups were compared by using the independent-samples t-test and *P*-values were calculated from this test. A *P*-value of 0.05 or below was accepted as statistically significant. In addition, Pearson’s correlation coefficient was used to evaluate the relationship between genes and miRNAs. The *P*-values for correlation coefficients were also calculated and reported in the table.

## Results

**Figure 1:**
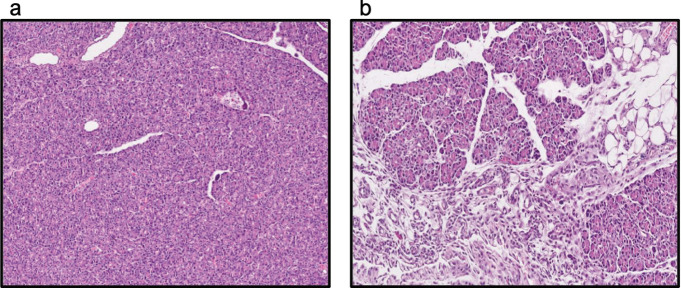
Hematoxylin and eosin (H&E) staining of pancreatic tissue samples. (a) Sham (pancreatic tissue with normal configuration and acinar structure) HE x100, (b) chronic pancreatitis (intense inflammation, glandular atrophy, and glandular edema in pancreatic acinar structures with adjacent fibrosis) HE x100

In our study, the MDA and TGF-β values in the CP group were observed to increase compared with the sham group (Table 3a). At the same time, histopathologic investigation showed the presence of inflammation, atrophy, and fibrosis in the CP group (Figure 1), and scores were identified to be significantly high compared with the sham group. The histopathologic appearance of the groups is shown in Table 3b.

**Figure 2:**
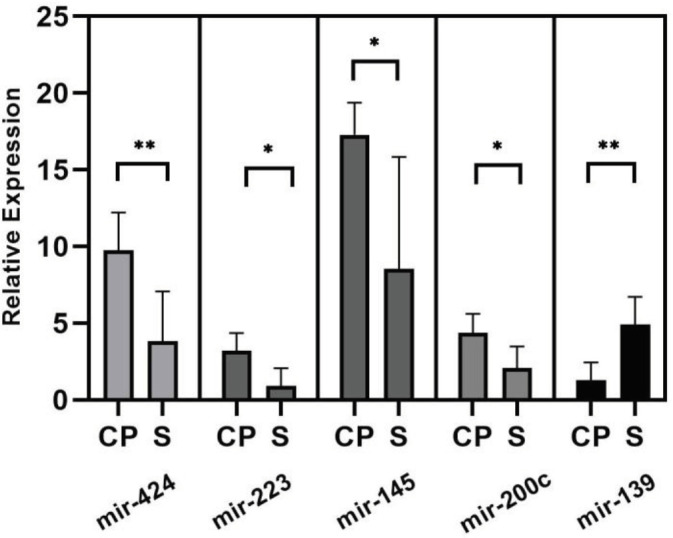
Relative expression levels of miR-223, miR-424, miR-145, miR-200c, and miR-139 in CP compared with the control, Data are expressed as relative fold change among data sets and each was performed in triplicate

According to our PCR results, the miR-139 expression levels in the pancreas tissue of the CP group were reduced by a clear amount compared with the sham group (*P*=0.003). Contrary to this, the expression levels of miR-200c (*P*=0.03), miR-145 (*P*=0.03), miR-223 (*P*=0.01), and miR-424 (*P*=0.005) were clearly increased compared with the sham group (Figure 2). When CP and sham groups are compared, there were no significant changes identified in miR-221 and miR-377 expression levels (*P*<0.5).

DIANA-miRPath v.3, miRWalk, miRPathDB, and HMDD v3.0 programs were used to identify miRNA-miRNA interactions on specific pathways. The 7 miRNAs chosen based on *in silico* analyses were identified to play roles in ECM-receptor interaction, pancreas cancer, endoplasmic reticulum protein process, and many known pathways (Table 2). 

The presence of inflammation in tissues with the induced CP model was tested with qRT-PCR variations in TNF-α and IL-6 gene expressions. As a result, TNF-α levels increased in the CP group (*P*=0.01), and additionally, IL-6 levels were identified to increase in the group with CP (*P*=0.02). In the CP group, *Ire1α* and *Perk* gene expression levels were observed to increase compared with the sham group (*P*=0.01 and *P*=0.06, respectively).

The variation in apoptosis levels was evaluated with qRT-PCR analysis of *Casp3* and *Bcl-2* genes. In line with the results, the CP group was identified to have a 2.5-time reduction in Casp3 and nearly 2-time reduction in *Bcl-2* compared with the sham group (*P*= 0.02, *P*= 0.007). In the CP group, the *catalase* and *α-Sma* expression levels were clearly increased and *Gpx1* gene expression level was clearly reduced compared with the sham group (*P*=0.007, *P*=0.01, and *P*=0.009, respectively). The correlation analysis results between miR-223, miR-424, miR-145, miR-200c, and miR-139 with *Bcl-2, Casp3, Cat, Gpx1, IL6, TNF-α, α-Sma, Ire1α,* and* Perk* gene levels are given in Table 4. Results show a positive correlation between miR-200c and miR-145 expressions with endoplasmic reticulum stress, oxidative stress, apoptosis, and inflammation levels, while there was a negative correlation with miR-139. Apart from miR-424, all miRNAs were associated with fibrosis, while miR-424 was only associated with genes for apoptosis and endoplasmic reticulum stress, and miR-223 was not identified to be significantly associated with endoplasmic reticulum stress. 

**Table 1:** Primer sequences used in qRT-PCR analysis

**Gene Name**	**Forward**	**Reverse**
***IRE1-*** ***𝛼***	CCTGAGGAATTACTGGCTTCTC	TCCAGCATCTTGGTGGATG
***Perk***	CGCTGCTGCTGCTGTTCCTG	GCAATGCCTCGGCGTCTTCC
***Casp-3***	ACTGGAAAGCCGAAACTCTTC	AGTTCCACTGTCTGTCTCAATA
***Bcl-2***	CGGGAGAACAGGGTATGA	CAGGCTGGAAGGAGAAGAT
***Catalase***	CAGATGAAGCAGTGGAAGGA	CAGGTGAGTTTGTGGGTTTC
***Gpx1***	CAGTTCGGACATCAGGAGAA	AGGGCTTCTATATCGGGTTC
***TNF-*** ***𝛼***	CCAGGAGAAAGTCAGCCTCCT	TCATACCAGGGCTTGAGCTCA
***IL-6***	TCCTACCCCAACTTCCAATGCTC	TTGGATGGTCTTGGTCCTTAGCC
***Gapdh***	TATCGGACGCCTGGTTAC	CTGTGCCGTTGAACTTGC
***𝛼*** ***-Sma***	TTCCAGCCTTCCTTTATCG	TTGGCGTACAGGTCCTTC

**Table 2:** KEGG pathway analysis of selected miRNAs

**KEGG pathway**	***P*** **-value**	**# ** **miRNA**
ECM-receptor interaction	6,84E+10	6
Hippo signaling pathway	2,72E+01	7
Pathways in cancer	1,65E+05	7
Pancreatic cancer	3,10E+06	5
Protein processing in the endoplasmic reticulum	0.0032	7
Signaling pathways regulating pluripotency of stem cells	4,45E+06	7
PI3K-Akt signaling pathway	0.019	7
Regulation of actin cytoskeleton	0.001	7
TGF-beta signaling pathway	3,30E+06	6

CP: Chronic pancreatitis; S: Sham. ∗*P*<0.5, ∗∗*P*<0.1

**Figure 3:**
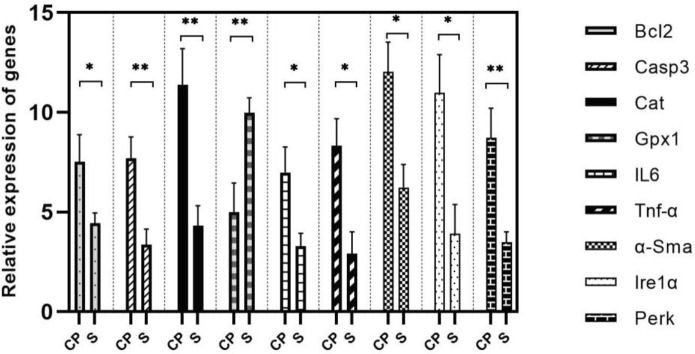
Relative expression levels of *Bcl-2, Casp3, Cat, Gpx1, IL6, TNF-α, α-Sma, Ire1-α,* and *Perk* in CP versus control. Data are expressed as relative fold change among data sets and each was performed in triplicate

CP: Chronic pancreatitis, S: Sham. ∗*P*<0.5, ∗∗*P*<0.1

**Table 3:** Distribution of biochemical and histopathological scores of study groups

		**Group 1 (CP)**		**Group 2 (S)**	**p**
**a.Biochemical results**	**MDA (nmol/ml)**		2.66±0.5	1±0.4	*P*<0.1**
	**TGF-β (ng/L)**		260.23±13.2	150.12±8	*P*<0.1**
**b.Pathology results**	**None**		0	7	*P*<0.1**
	**Mild**		1	0	
	**Moderate**		3	0	*P*<0.5*
	**Severe**		3	0	*P*<0.5*

A correspondence analysis was applied to determine which histopathological scores appeared more in which group. The difference between CP and sham group was significant *P<0.5, **P<0.1

**Table 4:** Correlation analysis between miR-223, miR-424, miR-145, miR-200c, miR-139 and *Bcl-2, Casp3, Cat, Gpx1, IL6, TNF-α, α-Sma, IRE1-α *and *Perk* in CP and sham groups of rats (R values)

	***Bcl-2***	***Casp3***	***Cat***	***Gpx1***	***IL6***	***TNF-***	***-Sma***	***Ire1-***	***Perk***
**miR-139**	-0,533	-0,750**	-0,513	-0,603*	-0,583	-0,714*	-0,665*	-0,593	-0,675*
**miR-200c**	0,667*	0,836**	0,580*	0,792**	0,733**	0,787**	0,623*	0,661*	0,818*
**miR-145**	0,575	0,893**	0,712*	0,944***	0,775**	0,776**	0,699*	0,642*	0,786*
**miR-223**	0,517	0,632*	0,532	0,663*	0,452	0,658*	0,651*	0,585	0,595
**miR-424**	0,256	0,713*	0,410	0,726*	0,466	0,468	0,329	0,338	0,531

∗*P*<0.5, ∗∗*P*<0.1, and ∗∗∗*P*<0.01

## Discussion

CP is a chronic disease characterized by fibrosis developing after long-term inflammation affecting pancreas functions irreversibly. The search for explanations of the physiopathology of this disease with high morbidity and causative treatments continues in the present day (13). In our study inducing a CP model with caerulein, parameters showing variations at biochemical, histopathologic, and molecular levels were assessed and we investigated the miRNA that we think plays roles in the physiopathology of the CP process after *in silico* analyses. MiRNA play a role in homeostasis of cellular activities like cellular development, differentiation, and cell death. Changes in the expression of miRNA were shown to be associated with pathogenesis in many diseases including inflammatory diseases and cancer (5, 7). 

In this study, of the chosen miRNAs, miR-200c, miR-145, miR-223, and miR-424 levels were significantly increased in the CP group compared with the sham group, while the miR-139 level was reduced. These results show that these miRNAs may act as biomarkers for CP diagnosis. The results show that the deregulation of miR-200c, miR-145, and miR-139 in CP is effective on endoplasmic reticulum stress, oxidative stress, apoptosis, and inflammation. The variations in expression of miR-223 and miR-424 in the presence of CP were shown to play roles in apoptosis and endoplasmic reticulum processes. Previous studies showed that miR-145 clearly increased in the presence of oxidative stress in HUVEC cell lines (14). It is reported that apoptosis, inflammation, and oxidative stress are triggered by the miR-145-5p/NF-κB pathway in human pulmonary microvascular endothelial cells (15). On the other hand, miR-139 was stated to cause significant reductions in oxidative stress, destroy unrepaired DNA damage and suppress apoptosis in many chronic diseases and cancer (16-18). For example, Deng *et al*. identified a panel of eight-miRNAs that would serve as early diagnostic biomarkers for pancreatic cancer (19). In that mentioned study, they showed that miR-139 significantly decreased in the case of pancreatic cancer. Another study reported that in the presence of airway inflammation in asthma, hsa-miR-223 regulated the TLR/Th17 signal and endoplasmic reticulum (ER) stress (20). Additionally, miR-223 was reported to be intensely expressed in the ER membrane of human brain tissue (21). There are studies reporting that miR-223, miR-200c, miR-145, miR-139, and miR-424 play roles in cell apoptosis and regulate apoptosis in pancreatic cancer (22-26). 

In a CP mouse model induced with high-dose ethanol, there were increases in ER stress (ATF6, PERK, eiF-2), oxidation of proteins showing oxidative stress, and iNOS expressions (27). Clinical and animal studies of CP reported the increase in chronic oxidative stress was responsible for clinical symptoms and histologic findings (28). miR-200b and miR-200c expressions in serum exosomes in patients with pancreatic ductal adenocarcinoma were shown to be much higher compared with healthy controls and patients with CP (29). 

In the literature, there are limited numbers of studies about potential biomarkers to be used for CP, and the number of studies investigating the role of miRNA in this topic is inadequate (30). In the literature, our study is the first to induce an experimental CP model in rats and research the role of miRNA identified with *in silico* analysis. Our results show that all five miRNAs (miR-139, miR-200c, miR-145, miR-223, and miR-424) are candidate biomarkers that may be able to determine the CP process.

A limitation of our study is the need to confirm miRNA expressions in large patient cohorts with CP diagnosis to confirm the specificity of the miRNA undergoing changes.

## Conclusion

miR-139, miR-200c, miR-145, miR-223, and miR-424 play roles in CP pathogenesis, and these miRNAs may be used as noninvasive biomarkers for detection and diagnosis of this disease. Additionally, the correlation between miR-200c, miR-145, and miR-139 with endoplasmic reticulum stress, oxidative stress, apoptosis, and inflammation may provide information about the prognosis of CP and the detailed effect of specific treatment choices related to causes. 

## Authors’ Contributions

EGT, Study conception and design; data analyzing and draft manuscript preparation; critical revision of the paper; supervision of the research and final approval of the version to be published.

## Conflicts of Interest

The author have no conflicts of interest.
